# The psychological and subjective experience of catatonia: a qualitative study

**DOI:** 10.1186/s40359-022-00885-7

**Published:** 2022-07-15

**Authors:** Zukiswa Zingela, Louise Stroud, Johan Cronje, Max Fink, Stephan van Wyk

**Affiliations:** 1 Executive Dean’s Office, Nelson Mandela University, Gqeberha, South Africa; 2 Department of Psychology, Nelson Mandela University, Gqeberha, South Africa; 3 Department of Psychiatry, Stony Brook University, New York, USA; 4 Department of Psychiatry and Human Behavioural Sciences, Walter Sisulu University, Mthatha, South Africa

**Keywords:** Catatonia, Psychological, Subjective, Fear, Anxiety, Withdrawal, Obedience

## Abstract

**Background:**

Catatonia is a severe psychomotor disorder that presents as abnormality of movement which may also be excessive or severely slowed. It often inhibits communication when protracted or severe. In this study we investigated the emotive and cognitive experience of patients with catatonia during a prevalence study in an acute mental health unit from August 2020 to September 2021. The value of this study is the addition of the inner and often unexplored cognitive and emotive experience of patients in the description of the catatonic state, which lends an additional dimension to complement the medical and psychopathological descriptors that have been the focus of most studies on catatonia.

**Methods:**

Ethical approval was received from the Nelson Mandela University Human Research Committee and convenience sampling was undertaken to recruit participants admitted into an acute mental health unit with catatonia, four to eight weeks after discharge, following admission. The BFCSI and BFCRS and a pre-designed data collection sheet were used to assess n = 241 participants, and collect data on descriptions of thoughts, feelings, and behaviours they experienced during the catatonic episode.

**Results:**

Forty-four (18.3%) of the total 241 participants who were assessed had catatonia. Thirty (68.2%) of the 44 participants with catatonia provided data on their experience of catatonia. Twenty-three were males (76.7% of 30) and seven were females (23.3% of 30). All were within the age range of 17 to 65 years. The dominant themes of thoughts, feelings, and behaviors described centered around yearning for or missing loved ones, heightened fear, intense anxiety, negative affect, aggression, obedience, and withdrawal.

**Conclusions:**

The common themes that emerged from this study were overwhelming anxiety, fear, and depression. These were found to occur frequently in patients with catatonia when describing their psychological experience. These experiences may possibly relate to the flight, fight, freeze and fawn response, as described in prior studies on the subjective experience of catatonia.

*Trial registration*: Not applicable.

## Background

Catatonia is a psychomotor abnormality that presents mainly with prominent psychomotor abnormalities that include excessive movement, significantly decreased movement and abnormal movement [1–3]. It interferes with a patient’s ability to communicate, especially when an episode is severe, protracted, or characterized by prominent mutism, excitement, or speech oddities [1]. This means patients are often unable to describe their experiences during an acute episode. In this paper, we explore the catatonic experience using self-reports collected during at follow-up from participants who screened positive for catatonia. The prevalence of catatonia is wide, ranging from just below 10% to a little over 60% per year [2–5]. The prevalence rates at the study site were found to be 11.9% over 6 months and 18.3 per year [5]. Catatonia is often episodic with a good response to treatment, but it may also be protracted and potentially fatal if it is not detected early enough or if it is not treated effectively [3].

### The psychological experience of catatonia

Patients with catatonia describe experiencing very intense emotional states. In the Northoff et al. [6] paper on the subjective experience of catatonia, 24 patients with catatonia provided self-reports at the 3-week point following an episode of catatonia. Their experience was less focused on the change in their movements and more on the change in their cognitive or affective experience. This included intense emotions that could not be controlled. There was also ambivalence, which correlates with one of the signs that may be observed in catatonia, namely ambitendency. This is the motor manifestation of being caught in two opposing actions at the same time. Northoff et al. [6] went on to categorize domains for assessment of the subjective experience of catatonia, which included the patient’s subjective description of their altered motor function, their interaction with their environment, and their emotional and cognitive experiences during the catatonic episode.

Rosebush and Mazurek [7] found that most patients felt very anxious during the catatonic episode, and 15% thought they were either already dead or were going to die. Shorter and Fink [8] and Moskowitz [9] characterized catatonia as a state of extreme fear that manifests as freezing, like that seen in some animals who respond with immobility or freezing when faced with danger. Shorter and Fink [8], address this in their book on catatonic stupor, The Madness of Fear, and ask whether catatonia is a mostly a neurological phenomenon with no psychological explanation behind it or whether it is a case of the mind remaining active during catatonia, even though one is unable to communicate.

From the 1900, catatonia was postulated to have a psychology of its own with symptoms seen as symbolic of underlying psychological drivers [8]. Around the same time, Wernicke proposed the concept of motor psychosis which was understood to view catatonia as “abnormalities of motion and of speech which were independent of thought or will” [8]. Northoff postulated that it was the intensity of emotions, whether positive (as in catatonic excitement) or negative (as in catatonic stupor), that triggered catatonia [6].

Fear or a heightened state of anxiety have also been described during a catatonic episode [6–12]. This intense anxiety may be observable and accompanied by a sense of impending doom with the patient feeling that they will die. Some patients may even believe that they are already dead [12, 13].

Based on the limited studies available on patient reports about the catatonic episode, the main psychological drivers and subjective experiences of catatonia have been around intense negative emotions like fear and heightened anxiety. Novac et al. [13], postulate that one may look at catatonia from an evolutionary perspective, meaning, this is a survival mechanism, applied when there is perceived impending danger. This is similar to what Northoff [1], Shorter and Fink [8], Rosebush and Mazurek [9], and Moskowitz [10] also postulate. So, although there is still scanty literature on the psychological impact of catatonia and the subjective experience as described by patients, what is available does seem to implicate fear circuits and somehow involves the perception of immediate danger or threat that the patient perceives.

To understand this better, and also how catatonia might fit into this theory of being fear- and danger-driven, one can compare the response to fear in humans to the response seen in animals. Whether in humans or animals, it may manifest as the fight-flight-freeze response that has been described as a built-in defense mechanism triggered by fear or trauma, whether physical or psychological. Walter Cannon, a medical doctor and physiologist who studied the stress response, first described this observation back in 1915, as quoted by Quick and Spielberger [14].

Cannon’s research and pioneering work on stress and how it affects the human psyche and physical state, served to advance the study of psychosomatic medicine, highlighting how a persons’ psychological state could affect their physical health [14]. He also highlighted the response to stress and how it can drive behavior, including how danger, or conditions that generate a stress response, can evoke heightened feelings of anxiety and anger accompanied by an autonomic nervous system reaction with all its involuntary bodily changes [15]. This then drives the fight-or-flight response. Cannon’s work has been studied and expanded upon over the years and this reaction is now recognized to potentially also include the freeze-and-fawn responses, which are also linked to the same defense system against fear- or anxiety-inducing stimuli [16]. This fear reaction is explained below:

- Fight—this entails facing the danger and fighting the threat aggressively.
- Flight—this implies running away from the threat to try and save yourself.
- Freeze—this is equivalent to playing dead through immobility until the threat passes.
- Fawn—This is the submissive response which serves to avoid conflict [15, 16].

### Relevance of catatonia to psychology

The medical approach to treatment emphasizes biopsychosocial interventions. Interventions for catatonia, however, are solely biological until the acute catatonic episode has settled. Psychological interventions are usually reserved for intervention until after the acute catatonic episode. Interventions like specific psychotherapies are then tailored to the underlying condition driving the catatonia.

If one accepts that the drivers of the catatonic response include fear or extreme anxiety, then the signs of catatonia may be recognized as the range of responses one gets in response to danger, as described by Cannon and expanded on by others [15–17]. Signs of catatonia, like withdrawal, immobility, and stupor, may be equivalent to the freezing aspect of the stress response; negativism and combativeness to the fight response; and automatic and passive obedience to the fawn reaction.

Based on the evidence by Northoff [1], Fink and Taylor [3, 17], Rosebush and Mazurek [7], and Moskowitz [9], one expects reports of fear and anxiety with different aspects that fit into the fight, flight, freeze, or fawn response to be at the forefront of participants’ self-report. The design of the enquiry undertaken in this part of the **study** was qualitative and exploratory so as not to lead participants but to rather let them set the themes for the emotive, cognitive, and behavioral experiences and responses during the catatonic episode.

Some studies have investigated catatonia in association with dissociation and post traumatic stress disorder (PTSD) [18, 19]. Ross and Browning [18], investigated an association between catatonia, dissociation, and adverse childhood experiences (ACE) and found a significant correlation between catatonia and adverse childhood experiences (ACE) than to dissociation. In their review on catatonia and PTSD, Biles, Anem and Youssef also postulated a possible correlation between psychological trauma and catatonia, based on the following: episodes of intense fear associated with trauma, PTSD resulting from the trauma and the associated responses of the PTSD which may be akin to catatonia [19].

Furthermore, data from the studies by Northoff [6] and Shorter and Fink [8], suggests that patients are aware of their emotive and cognitive experiences during the non-communicative stage of acute catatonia. In a retrospective study on 24 patients who had experienced catatonia, Northoff described reports of ambivalence and intense emotions which could not be controlled. One could therefore speculate that exploration of the benefit of supportive psychological interventions like those targeted specifically towards re-assurance and those focused on helping to manage overwhelming fear and anxiety, may be worth further research.

### Aim

This current study was conducted as a complementary part of an overall study on prevalence of catatonia [5]. The aim was to explore the psychological and subjective experience of catatonia by collecting data on participants’ description of what their thoughts, feelings and behaviours were during the catatonic episode.

## Methods

### Study design

This part of the study used a prospective qualitative exploratory method to collect descriptions of the cognitive, emotive, and behavioral changes that participants experienced during the catatonic experience four to eight weeks after discharge.

### Setting

The study was in an acute mental health unit within a regional hospital in Nelson Mandela Bay in the Eastern Cape, a metro with a population of 1.2 million people [21]. Challenges like unemployment, poverty, and substance abuse, contribute to the mental health disease burden in the region [22]. Referrals to the **mental health unit (MHU)** are from departments like Internal Medicine, Accident and Emergency, Obstetrics and Gynecology, Pediatrics and Child Health, and Family Medicine in the hospital. The MHU has 35 beds with a multidisciplinary team that offers assessment and interventions, including electroconvulsive therapy.

### Study process

The study was conducted in accordance with the relevant institutional and national guidelines. Further information on ethics approval of the study is provided under the section, “Declaration”, below. All patients diagnosed with catatonia in the mental health unit were reviewed at the psychiatry outpatient clinic four to eight weeks after discharge. Participants were assessed by the research team, using the Bush Francis Catatonia Screening Instrument (BFCSI) and the Bush Francis Rating Scale (BFCRS) and DSM-5 (Diagnostic and Statistical Manual-5) and the follow-up section of the data collection sheet used for the catatonia prevalence study, to screen for recurrence of catatonia. Any patient who screened positive for recurrence of catatonic symptoms was referred back to the treating team for further management. The possibility of adding a control group of participants was explored to enable determination of whether the reported subjective experiences could be attributable to catatonia specifically. Given that the relevant questions on experiences during the admission period required patients to link their experiences to the catatonic episode specifically, it was decided to forgo this initially and to first conduct an on observational exploratory study, the intention being to use the findings to guide the development of improved study methods and more focused study questions in a follow up study.

### Participants, sampling, inclusion and exclusion criteria

Convenience sampling was conducted to recruit patients who had consented to take part in the catatonia prevalence study [5]. Patients who had screened positive for catatonia and had been admitted during the study period from September 2020 to end of August 2021, were recruited for the current qualitative arm of the study. Participants who refused consent at any point of data collection were excluded as well as any participants whose catatonia had not resolved due to the limitations with communication that are often associated with active catatonia.

### Assessment tools

The BFCSI and BFCRS and a pre-designed data collection sheet were used to assess participants and collect data. The BFCRS assessment was done once on admission and once at follow up. The Bush Francis Catatonia Screening Instrument (BFCSI) is comprised of the first 14 items of the BFCRS scale and was developed for use as a screening tool for catatonia (20, 21). The full Bush Francis Catatonia Rating Scale, which includes items 15–23 on the scale, was developed as a complementary tool to be applied when two or more of the first 14 items on the BFCSI are found. Appendix E shows the BFCRS and the instructions on use of the scale.

In the data collection sheet, participants were required to describe their experience during the catatonic episode through providing an account of the experience in their own words, as well as what their thoughts, feelings, and behavior were during the catatonic episode. The following statement was posed to them: “Please describe, in your own words, your experience of the catatonic episode in terms of your thoughts, feelings and behaviour”.

### Data management and analysis

The study took a qualitative narrative approach to explore the subjective experience of catatonia through interviews conducted with participants who had catatonia and field notes recorded during the outpatient assessments to gain an in-depth description of the participant’s experience of catatonia. This is an approach that collects stories about people’s experience or life events; in this instance, the focus was the lived experience of catatonia which was severe enough to require hospitalization [23, 24].

A thematic analysis of the data was conducted using the Braun and Clarke approach which has also been supported by others with themes identified through word repetition, metaphors, similarities, and keywords [25–27]. This process contributed to establishing a tentative hypothesis and theory regarding the emotive, and cognitive experience of catatonia in this cohort.

**Fig. 1:**
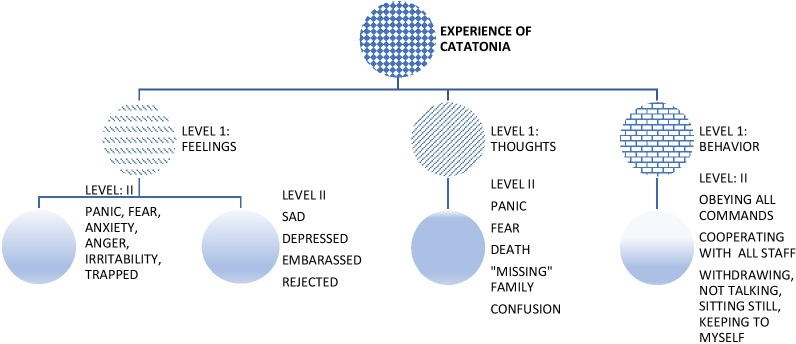
Decision Tree for feedback on participant experience during a catatonic episode

The data were analyzed in two sessions. For the first session, the researcher, assisted by two research assistants, organized the data into categories of thoughts, feelings, and behaviors of all 30 participants pooled together. Further analysis as guided by the consolidated criteria for reporting qualitative research (COREQ) was undertaken [28]. A decision tree on the processing and categorization of participant responses is reflected in Fig. 1.

In the second session we analyzed the three categories of feedback further to reach a consensus on the interpretations of keywords, similarities, repetition, and metaphors. Further, we looked for linguistic emerging themes. The process was repeated once more until data saturation was achieved on all three categories.

## Results

There were 241 participants who were screened for catatonia over the twelve months of the study. Of the total sample of 241 participants, 44 (18.3%) of them screened positive for catatonia on the BFCSI. Up to thirty of the 44 participants who screened positive for catatonia were successfully recruited for the follow-up qualitative arm of the study. Fourteen (31.8%) of the 44 were still admitted and were therefore excluded from the follow-up assessment as guided by the exclusion criteria.

We found that all participants used accessible, everyday language for their descriptions, which made it possible to reach consensus on the meaning of words or phrases used. Thus, we were able to compile a consensus list in each category, which highlighted keywords and themes.

### Demographic and clinical data

Of the 30 participants, 23 (76.7%) were male and 7 (23.3%) were female. The age range was from 18 to 65 years with an average age of 31 years (standard deviation: 12.5). The majority of participants were Black (28 = 93.3%), with two (6.7%) identifying as Mixed Race. Schizophrenia was the most common diagnosis in the 30 participants (14: 46.7%) followed by bipolar disorder (9: 30%). The rest had a diagnosis of substance induced psychotic disorder, substance induced mood disorder or major depressive disorder. Up to 18 participants had substance misuse, with the commonest substance of abuse on self-report being cannabis (13: 43.3%), followed closely by alcohol (9: 30%). Lastly, the commonest presenting signs of catatonia in this cohort were found to be staring (30%), mutism (25%) and rigidity (17%) followed closely by stupor (15%). Table 1 provides a summary of important demographic and clinical data.

**Table 1:** Clinical and demographic data

Clinical/demographic data	No. of participants	Minimum	Maximum	Mean	SD
Age	30	18	65	31	12.5
Length of admission (days)	30	9 days	290 days	84.9 days	54.36 days
Vitamin B12 level	30	82 pmol/ml	545 pmol/mml	53.7 pmol/l	130.90
Creatinine Kinase level	30	32 u/l	1703 u/l	289.2 u/l	346.52
Iron level	30	1.3 mcg/dl	33.7 mcg/dl	12.7 mcg/dl	7.55
BFCRS Scores on admission	30	2	19	7	4.35
BFCRS Scores at follow-up	30	0	0	0	0
Sex	30	Male	Female		
		23			
Ethnicity	30	Black	Mixed race		
		28			
Previous admission	30	Yes	No		
		19			
Loss of a loved one?	30	Yes	Unknown		
		2			

### Thoughts

Up to 24 (80%) of the 30 participants expressed a preoccupation with thoughts of missing close family members or loved ones and a yearning to be with them. More often than not, these would be mothers or grandmothers. Some of the missed family members or close loved ones (a best friend in one case) had passed away close to the time of admission. Table 2 shows some of the thoughts described by participants which they had during the catatonic episode.

**Table 2:** Thoughts experienced during a catatonic episode

Description of thoughts
“Missing my mother”
“Missing my mother and my family”
“Missing my family”
“Missing my grandmother who passed away”
“Missing my grandmother and my kids”
“Thinking of my home, my father, my mother, and my family”
“I was wondering why I was there”
“I was wondering why I was admitted and not with my family”
“Death. Not caring about anything, even my children. Not knowing what was to come next”
“Thinking of my mother; thinking that something bad was going to happen”
“I was asking myself why I was here in hospital”
“They are coming to attack me”
“I will be killed”
“They are avoiding me” (other patients and staff)

The six (20%) participants who did not describe missing family members or yearning for them described a preoccupation with fear or anxiety (five of the six participants), death (two of the six participants), and loneliness (one participant). Five (16.7%) of the participants described that they did not know what was happening or were confused about what was happening to them. This implied a possible lack of awareness of just how sick they were and what their symptoms were.

### Feelings

The most commonly recurring feeling described by participants was that of deep sadness or depression in 13 (43.3%) of the 30 cases, and a feeling of yearning for or missing family members and loved ones. The sadness or depression was described even in cases where the diagnosis of the underlying condition was not indicated as depression or appeared to be mainly psychosis or mania. Next, in seven (23.3%) of the 30 participants, feelings of fear or feeling very anxious or panicky were described. Some patients described feeling both deep sadness and fear. When compared to thoughts, only five participants described experiencing a preoccupation with fear in their thoughts, but when correlated to the feelings described, up to seven of the participants described feeling very scared during the catatonic episode.

Other recurring themes included feeling very lonely (two participants), lost (two participants), embarrassed (one participant), or feeling a sense of missing their mothers mostly and, to some extent, their grandmothers (9). One participant described a feeling of rejection and three (10%) participants described feelings of extreme anger or irritation. Three (10%) participants indicated feeling trapped, with one participant stating that he felt like he was in prison. There were also participants who described feeling lonely (three participants; 10%) or withdrawn (two participants; 6.7%). Participant descriptions of feelings during the catatonic episode are reflected in Table 3.

**Table 3:** Feelings during an acute catatonic episode

Description of feelings
“Not safe”
“I was scared”
“I felt sad”
“I was depressed”
“Very anxious”
“Panicky”
“Lonely”
“Embarrassed”
“Rejected”

### Behaviors

Some participants were vague about how their behavior was affected during catatonia, with some indicating they could not remember their behavior. The most frequently described behaviors were cooperative (9; 30%), followed by obeying commands of treatment team (seven participants; 23.3%), sitting still or withdrawing (six participants; 20%), and being restless and or shouting and angry (three participants, 10%). Two (6.7%) participants described that they ignored everything and everyone and another said he could not move nor express himself even though he wanted to. Table 4 describes some of the behaviors reported by participants that occurred during the catatonic episode.

**Table 4:** Behavior during catatonic episode

Description of behavior
“I followed the staff commands”
“I cooperated with everything”
“Sitting still”
“I kept to myself”
“Kept very quiet”
“Sat still and keeping to myself”
“Not talking about anything”
“Not able to express myself”
“Cooperating and keeping to myself”
“Restless and shouting”
“Obeying. Doing what I was asked”

### Summary of findings

Thirty (68.2%) of the 44 participants with catatonia provided data on their thoughts, feelings, and behaviors during the catatonic episode. The dominant themes across thoughts, feelings, and behaviors centered around yearning for/missing loved ones with heightened fear, anxiety, and negative affect and associated behaviors being characterized by withdrawal and overly cooperative (submissive) behaviors. Other themes included feeling trapped, embarrassed, or rejected, and a preoccupation with death. Table 5 provides a summary of the emergent themes as described by participants.

**Table 5:** Summary of emerging themes describing the subjective experience of catatonia

Summary of emerging themes from subjective descriptions of catatonia		
Thoughts	Feelings	Behaviors
Fear Yearning (Missing) Confusion/trying to make sense of things wondering about death	Fear/scared/panicked/anxious Sad and/or anxious Confused Trapped Embarrassed Rejected	Obeying commands Cooperating with the staff (submission) Withdrawn/keeping to self Sitting still Not talking

## Discussion

The commonest diagnoses in this sample were schizophrenia and bipolar disorder, both of which can present with prominent anxiety. Furthermore, the commonest manifestation of catatonia in this sample was staring, mutism, and stupor which may be seen as a manifestation of overwhelming fear or anxiety. The lack of a control group in this sample thus has limitations with regard to ascribing the overwhelming anxiety described by participants to catatonia only. However, the researchers did take steps to mitigate this limitation by making the statement that was posed to participants more specific, asking about the experience of catatonia rather than the general period of admission during the acute illness.

The lived experience of catatonia described by participants reflects main themes of heightened fear, anxiety, and withdrawal emerging from this explorative work. This echoes descriptions by other researchers who have also explored this aspect; specifically, that of a state of heightened fear and/or anxiety [1–5]. These descriptions have equated the catatonic state to the freezing seen in some animals when overwhelmed by fear. The stillness of movement described by some participants who were conscious of experiencing this change in their behavior during the catatonic state may also be seen in this context.

When one considers the diagnostic criteria of the DSM-5 for catatonia, or the positive screening items of the BFCSI (Bush Francis Catatonia Screening Instrument), then this subjective description matches the slowed movement or stupor which are central to the diagnosis of catatonia. Up to nine (30%) of the 30 participants described some form of stillness, withdrawal, or not talking. In the catatonia prevalence study conducted at the study site, mutism was observed in (27) 61% of the 44 catatonic participants and stupor in 16 (36.4%) of them [5]. When one compares these findings on the reported subjective experience of catatonia to the objective findings of the catatonia prevalence study, nine participants, i.e., 33% of the 27 with mutism or 56.3% of the 16 with stupor, had a conscious awareness of the changes in their interactions with others or in their movements.

Another aspect worth noting is that 16 (53.3%) participants described that they were cooperating or “following commands” of the nursing staff. Firstly, this could be viewed as submission (or fawning), a defense seen in some animals in reaction to an external fearful stimulus or threat. Adams [29] described this type of animal behavior first, decades ago and more recently Dixon [30] and Reddon et al. [16] have expanded on this idea by describing it as an aggression avoidance or “saving” defense which is against the cost that aggression could bring to the animal should it be attacked after failure to submit. Dixon also highlighted the link to psychiatric disorders, linking it to signs and symptoms of depression which may in themselves be akin to the withdrawal seen in catatonia. Indeed, one of the underlying causes of catatonia is depression. Animals can thus switch from offense (fight) to submission (fawn), a reaction which is thought to be modulated via the ventromedial hypothalamus in rats and is thought to be similar in primates. This is also in line with the conceptualization by Shorter and Fink [4] of catatonia as a defense adaptation when one feels under threat, whether real or perceived.

Secondly, “automatic obedience” and “mitgehen” (extreme automatic obedience) are some of the signs contained in the BFCRS which, when present, are indicators of severity [20]. These are not included specifically as diagnostic criteria for catatonia in the DSM-5. Again, when examining the results of this current study one notes that automatic obedience and mitgehen were objectively reported by assessors in two (6.7%) of the 30 participants screened for catatonia [5, 31]. Yet there were up to 16 participants (53.3% of the 30) who reported some degree of obedience when describing their subjective experience of catatonia, implying that even though these signs could be objectively observed in two participants, more participants experienced some form of obedience and held an awareness of this change in their behavior.

Having laid out the subjective experiences described by patients about acute catatonic episodes, one could hypothesize that the possible drivers of catatonia in this cohort might be the intense emotions like fear and anxiety they have described experiencing. This requires further research and if proven to be correct, it may mean that there could be a role for psychological interventions in the management of acute catatonia, especially in vulnerable or at-risk individuals. By combining this knowledge with the risk profile that one could build based on the findings of earlier studies conducted at the site on prevalence and risk factors of catatonia, one might be able to find applicability for psychological interventions that target the management of stress, anxiety, and fear. Catatonia responds to specific biological treatments like benzodiazepines and electroconvulsive therapy (ECT), irrespective of the underlying cause. Similarly, one could use psychological interventions in acute catatonia to help patients manage the intense negative emotions that accompany catatonia, irrespective of the cause. Such interventions could be specifically targeted towards anxiety and fear management techniques to help patients gain control over their reactions.

Since sadness features prominently along with a yearning for loved ones and family members, this highlights a possible target area for intervention where the treating team could work with family and loved ones to support patients with catatonia. This could include running psycho-education sessions for patients and relatives, which may be complemented with short, well-timed family visits to maintain ongoing contact with loved ones even in the face of acute symptoms. This should be possible for patients with the retarded form of catatonia, especially if the relatives are willing to take part in some of the basic physiotherapeutic exercises that patients often must undertake to prevent the development of deep vein thrombosis or contractures. Cognitive strategies that target fear and anxiety could also be offered earlier in the treatment of catatonia.

### Significance of findings and clinical applications

The design of the study was exploratory in nature, and did not attempt to answer questions about causality between the catatonic episode and the subjective experiences described by the participants. At most, one could speculate that the data presented in the current study on emergent themes of overwhelming anxiety, fear and negative affect in the presence of catatonia, may allow one to speculate that there may be value in offering psychological interventions during an acute episode of catatonia. Such a hypothesis could be the focus of future, more targeted studies which could include a comparator control group to allow for more accurate interpretation of findings.

Understanding the subjective experience of patients with catatonia is important due to the value it brings to the direction of future research which may possibly lead to expanded interventions beyond the standard treatment for catatonia. Currently, the standard treatment for catatonia is medication like BZDs and also ECT, which have both been shown to be effective treatments [1, 3, 7, 10, 11]. There may therefore be unmet needs when it comes to the psychological interventions for catatonia. Current research reflects this in that there are no suggested psychological interventions for catatonia.

The findings in our study suggest the possibility of early introduction of behavioural and cognitive strategies as well as cognitive behavioural therapy (CBT) as soon as the catatonia has settled may be of some benefit in helping patients to manage the strong negative emotive experiences of catatonia. This is based on the current evidence of CBT for symptoms of anxiety, and it remains the psychologic treatment of choice for the majority of anxiety disorders [32–36]. This would also mean that instead of waiting until full remission of catatonia, which is the current practice, one would need to involve the psychology team in the multidisciplinary team management of the patient with catatonia earlier on. This could support the development of rapport earlier on and may be very useful in development of insight about the drivers of catatonic symptoms. As observed earlier, catatonia seems to be the one state where the psychological intervention is reserved until late into the condition.

Shah and Wing [30] suggested screening for precipitating stressors and targeting these through lifestyle and environmental changes coupled with stress reduction measures may help catatonic patients. These measures may be useful in the intermediate and long term but may not necessarily address catatonic symptoms in the acute stages since the nature of the presentation is such that communication is impaired. Even with the problems in communication however, some patients indicated a retained ability to follow what was going on around them.

### Recommendations

The data presented in this current study imply a sense of heightened noxious emotive and cognitive experiences which implies suffering and distress that seem to be prominent in patients with catatonia. A theoretical possible approach for non-pharmacological interventions for catatonia could be to focus on broad interventions that target the immediate relief of distress due to fear and anxiety. This is postulated to be one of the many reasons why benzodiazepines are so effective for catatonia since they are anxiolytic. Other theoretical supportive measures which may be of benefit during an acute catatonic episode are those targeted for reducing anxiety and hyperarousal, similar to interventions for patients with delirium [31, 32]. This is also suggested especially when considers the fact that catatonia can also occur in delirium [32]. Examples of such suggested which may be beneficial in theory are:

- nursing in a quiet environment.
- allocating core regular staff to manage the patient to allow for familiarity to develop relatively quickly.
- always adopting a reassuring, approach when interacting with the patient.
- providing well-timed, simplified, and regular feedback on the management plan (e.g., “Today is Tuesday. The doctor will review your treatment with you today,” “Today is Tuesday. Mary, the occupational therapist will be seeing you today,” or “Sipho, you seem to be having a better day this Tuesday” etc.).
- using language without jargon when talking to the patient re-enforcing orientation whenever possible.
- providing reasons for admission and a simplified plan for the day so that the patient has assurance about predictability to his immediate environment. See Table 6 for details on theoretical interventions which could be the focus for future research.
- As anxiety and fear settle, use behavioural/cognitive behavioural techniques to help the patient manage overwhelming emotions and thoughts.

**Table 6:** Subjective experience of catatonia and theoretical potential interventions for future research

Experience during catatonia	Theoretical Non-pharmacological interventions for future research
Fear and anxiety	Nursing in a quiet environment with low levels of potentially stressful stimuli
	Allocating regular staff to nurse the patient to allow for familiarity and recognition to develop relatively quickly
	Taking a reassuring, non-demanding approach to interacting with the patient during the catatonic state
	Emphasizing reassurance about safety of the environment and the caring aspect of the members of the multidisciplinary team involved in the patient’s management
	Open, non-threatening and reassuring demeanour at all times to help in reassuring patients
	Open sharing of all information regarding intervention plans for care using basic, easy to follow language to ensure there is no confusion. This should be done irrespective of whether the patient is communicative or not
Yearning for family	Psychoeducation sessions with family
	Short contact sessions for reassurance re ongoing family support. Increase contact with family and loved ones when aggressive outbursts decrease if patient displays any and explain this link to the patient as well i.e., it is for their safety and the safety of others
	Family mediated exercise interventions and support with activities of daily living (supported by a psychotherapist and occupational therapist)
Confusion about orientation and reasons for admission	Providing well-timed, simplified and regular feedback on the management plan (e.g., “Today is Tuesday. The doctor will review your treatment with you today”, or “Today is Tuesday. Mary, the occupational therapist will be seeing you today”, “Sipho, you seem to be having a better day this Tuesday”, etc
	Using simple, non-jargon language when talking to the patient and taking opportunities to re-enforce orientation and reasons for admission whenever they present themselves (as in the simple examples provided above)
	Reasons for admission and the plan for the day should be conveyed at every patient contact to help lend a degree of predictability to the patient’s immediate environment

### Strengths

This study presents data on the neglected and under-researched aspects of the psychological experiences of catatonia as described by patients. The process of data collection allowed patients to describe their experiences of the catatonic episode in their words, in order to minimize recall bias.

### Limitations

The qualitative method applied in this section collected subjective reports from patients one to two months after admission. Data collection therefore relied heavily on the participants’ ability to recall details of their admission, which had happened weeks prior to the interview. There is a possibility of bias due to the fact that recall may not always be accurate. The exploratory and qualitative nature of the study design which did not lead participants was a means to mitigate this potential bias. What was also striking, was the similarity in some of the experiences reported by participants, especially because the follow-up assessments were conducted on a one-on-one basis. Since many of the participants ended up describing the same cognitive, emotive, or behavioral manifestations in response to the open-ended questions and requests to share their experience, this lends a degree of reliability to the findings. Another limitation of the study is the lack of a control group which may have implications for the interpretation and generalizability of study findings because there is a likelihood that the described experiences may be attributable as much to the catatonic episode as to the underlying mental disorder (e.g., schizophrenia, bipolar disorder, major depressive disorder, etc.). Given previous similar reports in other studies on patients with catatonia, however, the authors speculate that there is likely a link between catatonia and these experiences. This speculation is further supported by the fact that the statement posed to patients about their experience was specifically directed at the catatonic episode (“Please describe, in your own words, your experience of the catatonic episode in terms of your thoughts, feelings and behaviour”). The question was deliberately worded to refer specifically to the patient’s experiences during the catatonic episode rather than the patient’s general experience during the admission period.

## Conclusion

This exploratory investigation into the subjective experience of catatonia has unearthed clinically relevant data on the cognitive, emotive, and behavioral aspects of catatonia. Additionally, it has identified themes of heightened fear, anxiety, and sadness with a tendency to withdraw from interactions with others and/or to become over compliant to external stimuli, a behavior postulated to be akin to the offense versus submissive defense employed by animals against aggression or threat.

## Data Availability

All data are stored at the study site and are available on request from the lead author at Nelson Mandela University, Executive Dean’s Office.

## Abbreviations

ACE: Adverse childhood events
BFCSI: Bush Francis Catatonia Screening Instrument
BFCRS: Bush Francis Catatonia Rating Scale
DSM-5: Diagnostic and Statistical Manual-5
MHU: Mental health unit
Stats SA: Statistics South Africa
